# Factors associated with interest in psychiatry in UK medical students: qualitative study

**DOI:** 10.1192/bjb.2021.71

**Published:** 2023-02

**Authors:** Grace Crowley, Sube Banerjee, Lisa Page, Stephanie Daley

**Affiliations:** 1 North Bristol NHS Trust, UK; 2 University of Plymouth, UK; 3 Sussex Partnership NHS Foundation Trust, UK; 4 Brighton and Sussex Medical School, UK

**Keywords:** Medical students, psychiatry, career interests, education and training, recruitment

## Abstract

**Aims and method:**

This study aimed to explore factors that positively influence UK medical students’ interest in psychiatry. Delegates and committee members of the National Student Psychiatry Conference 2018 were invited to participate in individual semi-structured interviews. Nine interviews were conducted. Qualitative data were analysed using thematic analysis.

**Results:**

Four core themes emerged: psychiatry education and exposure, role of a psychiatrist, fitting in, and factors external to medical school. All students had some degree of interest in mental health before medical school, but placement and extra-curricular factors were strongly influential.

**Implications:**

Interest in psychiatry may be promoted by facilitating student exposure to enthusiastic psychiatrists and psychiatry subspecialties, encouraging extra-curricular activities and identifying early those with pre-existing interest in mental health on admission to medical school. Aspects of psychiatry that should be promoted include the potential to make a positive difference to patients’ lives and the teamworking elements of the specialty.

There have long been concerns that more psychiatrists are required to meet the needs of the UK population.^1,2^ Psychiatry has traditionally been one of the least favoured specialties among junior doctors.^3^ As recently as 2017, under two-thirds of available posts for core psychiatry training were filled in the first round of applications, with a fill rate as low as 21% in the north-east of England.^3^ At consultant level, the number of vacant posts more than tripled between 2013 and 2019.^1^ Significant improvements have been made to core psychiatry recruitment in recent years, and 2020 saw a 99.4% fill rate.^4^ The reasons for this are not clear; however, contributing factors may include campaigns targeted at medical students, such as the Royal College of Psychiatrists’ (RCPsych's) *Choose Psychiatry* campaign,^5^ as well as broader influences such as government pledges to spend more money on mental health services, increased media coverage of mental health issues, and celebrities including the Royal Family discussing their own mental health problems. Maintaining recent progress in core training recruitment will be necessary to ensure a sufficient workforce in the coming decades. Beyond immediate recruitment, understanding interest in psychiatry may have implications in the medical workforce for promoting cross-specialty working, and respect for people with mental illness and the specialty itself.

Previous work in this area has largely focused on the medical student population as a whole, and the majority of studies have involved the collection of survey data.^6^ Studies that have collected qualitative data have been limited to analysis of text-based responses as part of surveys.^7,8^ Factors influencing interest and preferences in career choices are often complex and interrelated. A qualitative approach has potential to capture deeper insights into the factors influencing interest in psychiatry and identify more nuanced complexities that may not be fully captured by word-limited text answers within surveys. To our knowledge, this is the first qualitative study using individual interviews with UK medical students to identify the factors that have positively influenced their interest in psychiatry.

## Method

### Sample and setting

The sample consisted of delegates and members of the organising committee for the National Student Psychiatry Conference (NSPC) 2018, which was hosted at Brighton and Sussex Medical School (BSMS). In all, 122 tickets were purchased for the conference, with medical student delegates from 18 UK medical schools. Medical students who attended the conference (*n* = 82) were approached for inclusion in the study: 21 members of the organising committee and 61 delegates.

### Procedure

The study was introduced to delegates during an announcement at the NSPC 2018. Students were emailed after the conference asking for permission to send them further information about the study. Thirteen delegates gave permission to be sent the study information sheet. Of these, nine gave consent to participate in the study. A topic guide was developed following an initial review of the literature and was adjusted iteratively as the interviews progressed. The first participant was asked to pilot the topic guide and gave consent for their interview to be included in the analysis. Ethical approval was given by the BSMS research ethics committee.

Interviews were conducted by G.C. (a medical student/foundation doctor) and S.D. (a non-medical qualitative researcher) between April and October 2019. Participants were first asked to confirm their medical school and year of study, and to state how likely they were to choose psychiatry as a career on a five-point Likert scale from ‘Extremely unlikely’ to ‘Extremely likely’. The rest of the topic guide contained open questions and sought to explore the reason for attending the conference, factors underpinning interest in psychiatry, experiences of psychiatry (educational and personal) and, lastly, social representations of psychiatry. With the exception of the last topic area, social representations of psychiatry, all other topic areas were inductive. The final version of the topic guide is presented in the Supplementary material (available at https://doi.org/10.1192/bjb.2021.71). Interviews took place face-to-face or via video or telephone call, based on participant location and preference, and lasted between 18 and 50 minutes. All interviews were audio-recorded using an encrypted dictaphone and transcribed verbatim.

### Qualitative analysis

Transcripts were analysed using a process of thematic analysis, specifically surface coding. G.C. and S.D. independently reviewed one randomly selected transcript and allocated descriptive codes to meaningful segments of text. They then met to review their respective preliminary codes to identify areas of agreement and disagreement. The same process was performed for a second transcript. Following this, a draft coding framework was created and used by G.C. to undertake descriptive coding for the remaining transcripts. The software programme NVivo 12^9^ was used to organise codes and systematically review the data allocated to each code. G.C. and S.D. met several times to discuss refinement of codes and identify emerging core and sub-themes.

S.D. and G.C. also met on several occasions for reflection during the data collection and analysis period. This included reflection on the influence of G.C.'s own position (as a medical student/foundation doctor with an interest in psychiatry) on the study. In addition, G.C. and S.D. met with L.P. (a consultant psychiatrist/medical educator), to discuss emerging core themes and sub-themes with someone who was not involved in data handling.

### Ethics statement

This research was approved by the BSMS Research Governance and Ethics Committee (reference number: ER/BSMS3828/1).

### Consent statement

Participants were provided with a study information sheet and completed an electronic consent form.

## Results

### Participant characteristics

Nine participants took part in one-to-one semi-structured interviews: seven via video or telephone call and two in person. One had been a member of the NSPC organising committee and eight were conference delegates. Demographic data were missing for one participant. Of the remaining, there were six female and two male participants, with a mean age of 24 years. Five participants attended BSMS, two King's College London, one Bristol Medical School and one Leicester Medical School. Stage of training ranged from second year of medical school to having recently completed the fifth year. When asked ‘As it stands at the moment, how likely are you to choose psychiatry as a career in the future?’, two participants answered ‘extremely likely’, five ‘likely’, one ‘neither likely nor unlikely’ and one ‘unlikely’.

**Fig. 1:**
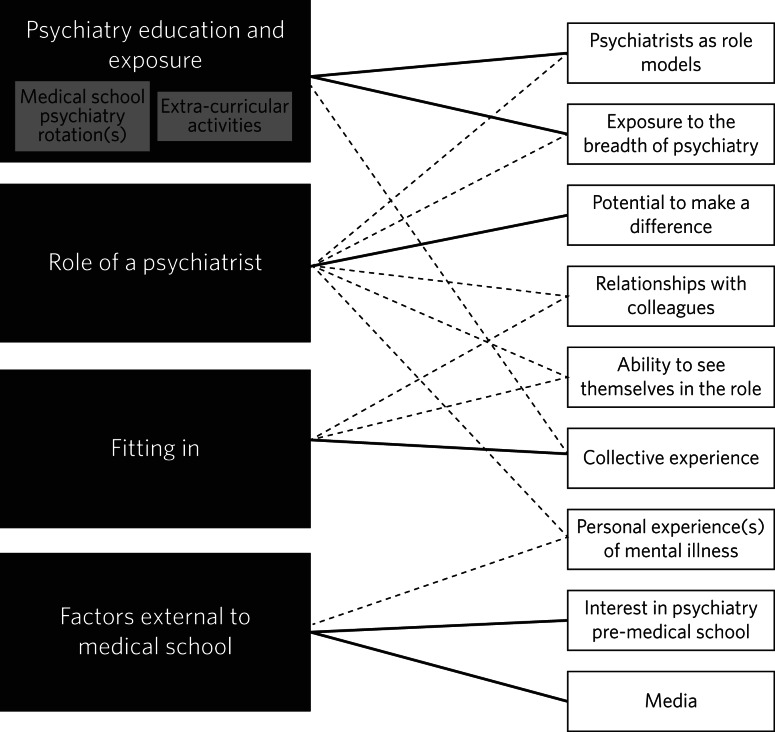
Visual representation of relationships between core themes and sub-themes. Solid lines represent relationships with the strongest evidence (based on number of participants who endorsed it).

We identified four core themes from the data; psychiatry exposure and experience; role of a psychiatrist; fitting in; and factors external to medical school. Nine sub-themes emerged, some of which related to more than one core theme. Figure 1 demonstrates the relationships between core themes and sub-themes.

Table 1 makes clear the wider content of each of the sub-themes, illustrated by example quotations, which are discussed in more depth below.

**Table 1:** Summary of sub-themes that emerged from interview transcripts

Sub-theme	Includes	Example quotations
Psychiatrists as role models	Psychiatrists’ enthusiasm for their job, encouraging medical students, admiring clinical acumen	**Participant 7:** ‘… a lot of doctors who are quite apathetic and almost like “Oh, why are you going into medicine?” whereas in psychiatry they were all very passionate about what they did, and that was quite noticeable I think’ **Participant 6:** ‘So, it gave me an appreciation for the skill involved which I don't think I'd had much of that before’
Exposure to the breadth of psychiatry	Awareness of subspecialties and career options gained through psychiatry placements or extra-curricular activities	**Participant 5:** ‘So, by doing some of these events and inviting these people and talking to those people, I've learnt there are a lot more kind of jobs in psychiatry and a lot more like variations of it than I realised’ **Participant 2:** ‘Yeah, I think it was good to see an area that isn't really covered in our sort of bog standard mental health placement, which is just your sort of general adult ward. I think that made it more exciting to see the different possibilities’
Potential to make a difference	Direct interaction with patients, potential to improve psychiatry services, shortage of psychiatrists	**Participant 5:** ‘I think just because I know that with psychiatry it is a lot more person orientated, it's more about kind of understanding people and kind of trying to help them in a particular way, and whereas pathology is kind of more looking at like cells and looking at tissue and stuff … and I just kind of want to like help people, just feel like doing psychiatry there's a more kind of direct way with that, because you are literally like face-to-face with people’ **Participant 9:** ‘… psychiatry is something that you could do so much good for people, that there is such a need for psychiatrists, there's a lack of psychiatrists, and it's an under-helped or under … lack of resources for people with mental health problems’
Relationships with colleagues	Supportive team environments, senior support for trainees	**Participant 7:** ‘… there's a lot of senior support and trainees are very much encouraged to escalate whenever they feel the need to, and I've never ever seen that being kind of frowned upon in the way that perhaps it is in other places’ **Participant 1:** ‘… everyone seemed to be very supportive of each other. So, it seemed like quite a nice environment to work in’
Ability to see themselves in the role	The type of person who chooses psychiatry, aspects of the role that appeal	**Participant 8:** ‘But attending the different workshops and also all the other workshops were held by different people who I could relate to, made me realise that potentially, oh, I am similar to those people, potentially I could do this’ **Participant 2:** ‘… made me realise that I quite like being in that supportive role and helping people to talk through things’
Collective experience	Being surrounded by others who also have an interest in psychiatry, having friends also interested in psychiatry	**Participant 5:** ‘I think it was nice just to see how many people were interested in it as well, because sometimes it can be a bit disheartening, like thinking that not many people want to go in to it …’ **Participant 7:** ‘… just the kind of like the people that were also there, the kind of people that are interested in psychiatry I always feel that I sort of get along with, like I think it probably matched my personality in that I find other people that are thinking about psychiatry are sort of similar to myself and that kind of thing. So, that's probably quite influential, yeah’
Personal experience(s) of mental illness	Experiences with their own mental health, experiences with family or friends’ mental health	**Participant 5:** ‘Probably just seeing … it's probably more to do with my friends, actually, like kind of seeing some of the stuff they were going through and stuff, and like it made me kind of think like ‘Oh, I could actually make like a real difference in terms of like being a doctor specifically in this area’ **Participant 3:** ‘… I really do sympathise and empathise with patients who do have psychiatric illness and after all my experiences: like, so I had a psychiatrist who I saw when I was in hospital and I was pretty sure that she'd never really been touched by psychiatric illness herself … and so I think hopefully that would improve my practice …’
Interest in psychiatry pre-medical school	Inherent interest, reading around the subject, school projects, Psychology A-Level	**Participant 6:** ‘Yeah, but it was a general interest, I've had since I was about 16 in how people's minds work …’ **Participant 2:** ‘… I did psychology at A-Level … and it was actually really interesting and particularly the module we did about schizophrenia really sparked my interest …’
Media	Increased presence in the media, subliminal messaging, wanting to challenge stereotypes	**Participant 5:** ‘And I think also at that point, like media attention, it was becoming a lot more like known and people were talking about it a lot more. So, it kind of, yeah, just made me realise that there was that potential there’ **Participant 4:** ‘And I guess, as well, in the media, you only really hear when things go wrong too, so I guess that influences it. Again, you would want to go in and be somebody who does it better’

### Psychiatry exposure and experience

Interaction with psychiatrists had a largely positive influence on participants' interest in psychiatry. Psychiatrists with enthusiastic demeanours and a positive outlook on work were commonly valued. On placements, students valued psychiatrists taking an active role in their learning and involving them in discussions about patient care and research. They also admired psychiatrists’ clinical acumen, especially their ability to communicate effectively with patients, listening skills and compassion. Even brief encounters with engaging psychiatrists, such as lectures or workshops, were influential in fostering participants' interest in psychiatry.

Participants reported that the range of subspecialties within psychiatry was appealing. Awareness of career options came from a variety of sources, including medical school placements, student selected components (SSCs), involvement in a psychiatry society or the NSPC. An awareness of academic psychiatry and research opportunities was also a positive influence. Extra-curricular activities which provided exposure to specialist or niche areas of psychiatry that they may not have experienced during medical school placements increased participants' interest. In addition, exposure to psychiatry through placement or extra-curricular activities was enhanced if students perceived themselves as being in a collective experience with others. For instance, a frequently reported positive aspect of the NSPC was that it brought like-minded people together and that meeting other students interested in psychiatry encouraged their own interest.

### Role of a psychiatrist

The most frequently reported aspect of the role of a psychiatrist that participants valued was the potential to make a difference. Participants felt that the patient-facing nature of psychiatry and the holistic approach allowed them to have a direct effect on patients, as opposed to other specialties where the effect on patients was perceived to be more removed. The level of demand for psychiatry and the potential to help large numbers of people as a psychiatrist was cited as important to some participants. There was also a perception that by pursuing psychiatry, they could make a further difference by supporting a specialty which has faced recruitment issues and is seen to be under-resourced compared with other areas of medicine.

The psychiatric working environment was generally appealing. Positive relationships with colleagues, cooperative teams and a sense that those working in psychiatry were satisfied with their jobs were highlighted as reasons for interest in psychiatry. In particular, the sense that doctors in psychiatry do not work alone, but that decisions are often made collectively with colleagues, was a perceived benefit. Through psychiatry placements, students identified that there was good senior support for trainees. As well as the nature of the work itself, participants described a heightened interest in psychiatry if they could envisage themselves in the role. Some developed this through personal experiences with mental illness; for instance, by supporting a friend through mental health difficulties.

When asked about their perception of other students’ and doctors’ views of psychiatry, participants felt that lack of understanding of the role and misconceptions about psychiatrists and psychiatry in general were barriers to others choosing psychiatry.

### Fitting in

As discussed, having collective experiences with others who like psychiatry served to enhance participants' interest. Participants were aware that many other students did not want to pursue psychiatry, so they found engaging with like-minded individuals encouraging. For some, partaking in extra-curricular activities allowed them to find other people they could relate to. There was a perception of a type of person who chose psychiatry. Having a sense of being ‘like’ others who were interested in or worked in psychiatry, and being able to envisage themselves in the role, contributed towards interest in the specialty.

### Factors external to medical school

All participants reported having had some degree of interest in mental health before they started medical school. For some this represented a general interest, whereas others had considered psychiatry as a career pre-medical school. Most were not able to attribute their interest to anything specific, describing an innate interest in mental health or, more broadly, the workings of the mind, which led them to engage in activities that further directed them towards psychiatry.

There was inconsistency around the importance of personal experiences of mental illness. Some students reported that the experience of supporting a friend or family member through mental health difficulties made them realise they could purse psychiatry. Others did not think their personal experiences had affected their level of interest in psychiatry.

## Discussion

To our knowledge, this is the first study to use individual qualitative interviews to explore the factors that have positively influenced interest in psychiatry among UK medical students. The most important factors were encountering psychiatrists considered to be role models, having an awareness of the range of subspecialties in psychiatry, benefiting from collective experiences with like-minded people, the potential to make a difference to patients, and having some degree of interest in the workings of the mind or mental illness pre-medical school.

An important finding of this study was that participants’ interest in psychiatry was enhanced through encountering enthusiastic psychiatrists who promoted the specialty and involved students in patient care. This complements previous work exploring choice of psychiatry^6,10–13^ and medical career choices more generally,^14^ which highlighted the positive influence of role models, and suggests that medical schools should pay particular attention not just to the psychiatry curriculum but also to the selection of psychiatry educators. Another key aspect was exposure to a range of psychiatry subspecialties at medical school, which has been identified previously and highlighted in the RCPsych's *Guidance for Medical Schools*.^6,11^

Previous qualitative research conducted with psychiatry trainees suggested that having psychiatry placements early on at medical school may lead to more students considering psychiatry.^13^ In our study, participants spanned year groups; some had not yet undertaken their psychiatry placement yet still expressed an interest in the specialty, indicating that there may be other ways of fostering interest outside the curriculum. A key influential factor among our participants was engagement in extra-curricular activities, which complements findings from several surveys.^6,11,12,15^ By employing a qualitative approach, we were able to investigate potential motivations for engaging in psychiatry-related extra-curricular activities, such as meeting positive psychiatrist role models at events, exposure to the breadth of psychiatry and having collective experiences with other students. A challenge with extra-curricular activities is that they may be criticised for ‘preaching to the converted’, i.e. only those students with some pre-existing interest attend. Innovative methods are required to attract students not currently interested in psychiatry to such events, such as running them during working hours or allowing them to contribute towards mandatory teaching such as SSCs. Another option is merging extra-curricular activities with other specialties to attract a broader range of students. Organisations such as the European Psychiatric Association promote the integration of physical and mental health as a stigma-reducing strategy to improve the image of psychiatry.^16^ This may help to increase understanding of the role of psychiatrists and reduce misconceptions among medical students, which our participants suggested may be barriers to others developing an interest in psychiatry. In addition, joint specialty and multidisciplinary events have potential implications not just for recruitment but also for increasing awareness of the overlap between physical and mental health, fostering professional respect and improving collaborative working.

Our findings highlight aspects of psychiatry that should be promoted to improve interest among medical students, namely the positive difference psychiatrists can make to patients and the strong culture of teamworking. Participants had seen good examples of senior support for trainees on their placements and spoke of the relatively flattened hierarchy of psychiatry compared with other medical specialties. One way of promoting these aspects of psychiatry is through media campaigns, which relates to our finding that exposure to television, film and other forms of media was influential for some participants. The RCPsych's *Choose Psychiatry* campaign has produced several videos which emphasise the positive effect that psychiatrists can have on patients’ lives.^5^ These videos could be included in medical school psychiatry teaching to ensure they reach all medical students and not just those with a pre-existing interest. In addition, more exposure to psychiatry trainees, as opposed to consultants, may serve to demonstrate good examples of the senior support and flattened hierarchy that emerged in our interviews. Trainee-student shadowing schemes were recommended in the RCPsych *Guidance for Medical Schools*.^11^

Interestingly, factors such as working hours, flexible work schedule and good work–life balance, which are considered influential for choice of medical specialty among medical students, were not reported as key factors in our study.^14^ However, we cannot deduce that these factors are not important, more that they may be generic factors and not therefore specific to psychiatry. In addition, our sample size may have limited the identification of wider factors.

Our findings in relation to the importance of ‘fitting in’ and acknowledging that there is a type of person who chooses psychiatry relates to the wider discourse around professional identity formation.^16–18^ Psychiatry is often viewed through a lens of vagueness and complexity, perhaps due to lack of agreement within the specialty on fundamental aspects such as biological versus social determinants of mental illness and use of diagnostic classification systems.^17^ Having clearer definitions of psychiatric diagnoses and the role of psychiatry within medicine may serve to further refine a picture of who a psychiatrist is and allow students to identify attributes which they can relate to. On the other hand, it may be argued that it is not possible or desirable for psychiatry to have clearer boundaries, and the focus should be on attracting people who can tolerate complexity, unknowns and ‘grey areas’. Certainly, there will be a proportion of students who admire psychiatrists because of their ability to do this, identify these attributes within themselves and consider working in psychiatry as a result.

All participants reported having some degree of interest in mental health pre-medical school. Interest in psychiatry on entry to medical school was found to be less common when surveying whole medical student cohorts, compared with our highly selected sample who were defined by their attendance at an extra-curricular event.^12^ However, our findings are in keeping with the results of an international survey which identified a strong association between interest in psychiatry among final-year medical students and pre-medical-school interest in psychiatry.^15^ Some participants reported having had a general interest in the workings of the mind or mental health but could not identify when or why this interest came about. It is difficult to design interventions to foster this type of innate interest, but identifying it early could enable focused approaches to encourage these individuals to seriously consider a career in psychiatry. Promoting the work being done to increase neuroscience content in the postgraduate psychiatry curriculum through the Wellcome Trust and Gatsby Foundation Neuroscience Project may also appeal to students with an innate interest in neurosciences.^19^ Other participants could pinpoint specific influencing factors, such as undertaking Psychology A-Level or a school project related to mental health, which are modifiable and could be promoted to increase interest in mental health among pre-university students. If medical schools wish to help improve recruitment to psychiatry, they could look to using pre-existing interest in mental health as a factor to consider at admission. Only a small number of medical schools currently accept Psychology A-Level as a second or third science subject;^20^ if this were more widespread, it could lead to a future workforce more primed towards psychiatry. Furthermore, identifying academically capable school students, with or without motivations to study medicine, and exposing them to interventions such as psychiatry summer schools may influence career choice, through raising awareness and promoting positive attitudes towards psychiatry.^21^

Finally, lived experience of mental illness was a factor for some of those interviewed. Given that mental illness is common among doctors and medical students,^22^ it is unlikely that this in itself fosters an interest in psychiatry. Supervision may have an important role in helping medical students and trainees who disclose mental illness to navigate career choices; Lawrence suggests that supervisors should be alert yet resist jumping to conclusions.^23^ There is an opportunity for further research in this area.

### Strengths and limitations

This study adds to the literature by examining the factors that positively influence UK medical students’ interest in psychiatry using individual qualitative interviews, which allows for more in-depth exploration than survey data.

There were several limitations, the most important of which is that this study involved a highly selected sample derived from medical students who were likely to have an interest in psychiatry owing to recruitment through the NSPC. There is, however, potential value in exploring a group with positive appraisals of psychiatry, since that was the focus of this project. We have focused on positive factors and there may be further factors in the general student population. Future studies in more representative samples would allow for further exploration of factors that enhance the attractiveness of psychiatry, as well as those that form a barrier to interest. It would be instructive to conduct a similar study, but of barriers, interviewing medical students who are not considering psychiatry or those who are avowedly negative in their views. Such data would enable a better understanding of these factors and the extent to which they may be modifiable.

Other limitations include the small sample size and the low representativeness of medical schools in the sample (with more than half of participants attending BSMS). Given the lack of qualitative research in this area, and the rich themes emerging, we considered that nine participants could be justified. However, it is recognised that other themes might have emerged with a larger and more diverse sample. We were not in a position to extend the study further; however, we do recognise that recruitment would have been enhanced had it taken place during rather than after the conference. A further potential limitation was recall bias, especially for questions related to interest pre-medical school. Finally, this research captured interest in psychiatry at one point in time; we recognise that attitudes may change both throughout and after medical school.

## Conclusions

This study identified factors that positively influence medical student interest in psychiatry. These can be broadly summarised as encountering enthusiastic psychiatry educators, exposure to the breadth of psychiatry subspecialties, attending events that allow a sharing of interest in psychiatry, and aspects of the role such as the potential to make a difference and a supportive teamworking environment. Potential implications include designing initiatives to promote psychiatry, which may in turn improve recruitment into psychiatry and raise awareness and respect among all medical students.

## Data Availability

Data are available on request by contacting the corresponding author.

## About the authors

**Grace Crowley** is an Academic Foundation Year 2 doctor, North Bristol NHS Trust, UK; **Sube Banerjee** is Executive Dean, Faculty of Health, University of Plymouth, UK; **Lisa Page** is a Clinical Senior Lecturer in Medication Education and Psychiatry, Brighton and Sussex Medical School, and Consultant Liaison Psychiatrist, Sussex Partnership NHS Foundation Trust, UK; and **Stephanie Daley** is a Senior Lecturer in Older People's Mental Health and Education, Brighton and Sussex Medical School, UK.

## Supplementary material

For supplementary material accompanying this paper visit https://doi.org/10.1192/bjb.2021.71. click here to view supplementary material
