# Supplementary material for: Factors associated with interest in psychiatry in UK medical students: qualitative study
Source: BJPsych Bull. 2023 Feb;47(1):48–55. doi: 10.1192/bjb.2021.71 (PMC10028548; doi:10.1192/bjb.2021.71)
Supplement: Supplementary file 1 [file S2056469421000711sup001.pdf]

Supplementary Materials

Supplementary Materials 1: Final version of the topic guide (version 4) used to direct semi-structured interviews

Background information

- What medical school do you attend?
- What year of medical school are you currently in?
- Please answer the following question: As it stands at the moment, how likely are you to choose psychiatry as a career in the future?

Extremely unlikely

Unlikely

Neither likely nor unlikely

Likely

Extremely likely

National Student Psychiatry Conference 2018

- Can you tell me what motivated you to attend the National Student Psychiatry Conference in February 2018?
- Did you gain anything from attending the conference?
- Do you think this directly influenced your preference for psychiatry?
- If so, what elements of the conference were especially key in influencing your preference for psychiatry?

Timeline

- Did you consider psychiatry before starting medical school?
- If yes, what do you think influenced that choice?
- If no, when did you know you wanted to do psychiatry, and what do you think influenced that choice?
- Did you previously have another preference for a different specialty/career?
- If yes, what was it and what changed?
- If no, why were you so certain that psychiatry was for you?

Previous degree/s

- Did you undertake a degree prior to starting medicine and/or an intercalated degree during medical school?
- If so, do you think this directly influenced your preference for psychiatry?

Involvement in extra-curricular psychiatry activities

- Besides attending the National Student Psychiatry Conference 2018, have you taken part in any other extra-curricular psychiatry-related activities or clubs? If so, what?

- What motivated you to get involved in these extra-curricular activities?
- Did these experiences directly influence your preference for psychiatry?

Personal experience

- Have you had any personal experiences of mental illness that you think may have influenced your preference for psychiatry?

Psychiatry placements at medical school

- How many psychiatry placements have you undertaken during your medical school training so far?
- How would you regard the quality of these placement/s?
- What did you think the working environment was like during your psychiatry placement?
- Did you find any good role models during your psychiatry placement/s?
- If so, what about them made them [a] good role model/s?
- Did these placement/s directly influence your preference for psychiatry?

Personality types

- Do you think there is anything about your personality type that has influenced your preference for psychiatry? If so, what?

Perceptions of psychiatry

- How do you think psychiatry as a specialty is perceived among medical students in general?
- How do you think psychiatry as a specialty is perceived among doctors in other specialties?

Social representations of psychiatry

- Have social representations of psychiatrists in the media, television or film influenced your personal preference for psychiatry?
- If so, in what way? Are there any platforms in particular that have influenced your preference for psychiatry (e.g. reading, news, social media)?
- Do you think social representations of psychiatrists in the media, television or film have an impact on the way medical students in general view psychiatry?
